# Supplementary material for: Does blockchain technology matter for supply chain resilience in dynamic environments? The role of supply chain integration
Source: PLoS One. 2024 Jan 5;19(1):e0295452. doi: 10.1371/journal.pone.0295452 (PMC10769029; doi:10.1371/journal.pone.0295452)
Supplement: S1 Appendix — (DOCX) [file pone.0295452.s001.docx]

**Appendix S1.**

Questionnaire items

| Blockchain technology: (1 = not considering it; 3 = considering it currently, which means that it has been planned for and is due to be implemented but not carried out yet; 5 = implementing successfully) |
| --- |
| BCT1. Smart contracts for automatically implementing multiparty agreements. |
| BCT2. Order validation and approval. |
| BCT3. Invoice processing and final payment settlement. |
| BCT4. Verification of conflict-free raw material. |
| BCT5. Validating the manufacturing parameters. |
| BCT6. Leveraging the scale of big data availability. |
| BCT7. Standardized quality documentation. |
| BCT8. Integrating manufacturing practices. |
| BCT9. Logistics asset management (such as machines, transport vehicles, warehouses, material handling equipment’s etc.) |
| Supply chain integration: (1= Strongly Disagree; 3=Neutral; 5=Strongly Agree) |
| SCI1. We use enterprise resource planning applications for integrating the different functions |
| SCI2. All the supply chain functions, namely: planning, sourcing, production, delivery and sales have data integration and are in synchronization with each other. |
| SCI3. We establish strategic partnerships with our key suppliers. |
| SCI4. We include key suppliers in planning and goal-setting activities. |
| SCI5. We use information technologies to share real-time information with our key suppliers. |
| SCI6. We involve suppliers in product design and development. |
| SCI7. We establish strategic partnerships with our key customers. |
| SCI8. We involve our key customers in improving inter-organizational processes. |
| SCI9. We create an environment of trust with our customers. |
| SCI10. We involve customers in product design and development. |
| Environmental dynamism: (1=very stable; 3=about average for all industries; 5= very volatile). |
| ED1. The rate at which your competitors’ products change. |
| ED2. The rate at which your firm’s products change. |
| ED3. The rate at which your supplier’s capabilities change. |
| Supply chain resilience: (1= Strongly Disagree; 3=Neutral; 5=Strongly Agree) |
| SCR1. Our firm’s supply chain is able to appropriately respond to unforeseen disruptions by quickly restoring its product flow. |
| SCR2. Our firm’s supply chain is well prepared to deal with the financial outcomes of supply chain disruptions through alternative plans. |
| SCR3. Our firm’s supply chain is able to maintain a desired level of control over structure and function at the time of disruption. |
| SCR4. Our firm’s supply chain has the ability to extract useful knowledge from unexpected disturbances, and utilization. |
