# Supplementary material for: Does blockchain technology matter for supply chain resilience in dynamic environments? The role of supply chain integration
Source: PLoS One. 2024 Jan 5;19(1):e0295452. doi: 10.1371/journal.pone.0295452 (PMC10769029; doi:10.1371/journal.pone.0295452)
Supplement: Attachment — Submitted filename: Response to Reviewers.docx [file pone.0295452.s003.docx]

Re-Submission to PLOS ONE: *Does blockchain technology matter for supply chain resilience in dynamic environments? The role of supply chain integration*.

| **Responses to Reviewer 1** | | |
| --- | --- | --- |
| **Comment** | **Response** | |
| Dear Reviewer,  Thank you very much for your review of our work and the provision of constructive comments and feedback. We hope that our modified paper has addressed your concerns and benefited from your comments and feedback to improve the quality of research reported in the paper.  Best regards | | |
| - The sentence "By enabling the sharing of information, internal integration plays a crucial role [77]" (before hypothesizing H3) has too old citation (2012) since the up-to-date paper prefers the citation between 2019-2023. You may replace this citation "Aunyawong, W., Wararatchai, P., & Hotrawaisaya, C. (2020). The influence of supply chain integration on supply chain performance of auto-parts manufacturers in Thailand: a mediation approach. International Journal of Supply Chain Management, 9(3), 578-590." because this paper depict the effects of supply chain integration on resilience (flexibility), especially in automotive industry. | | Many thanks for your suggestions. Based on your feedback, the respective reference has been updated. [Location of change: Section 2.3, page 5]. |
| - In 3.1 Sampling and Data Collection, The word "logistic manager" is incorrect, "logistics manager" is correct. | | According to your suggestion, the word has been changed to become “logistics manager”. [Location of change: Section 3.1, page 7]. |
| - In 3.2 Measures, the numbers of items should be specified. (for example, 4 items of SCR were derived from...) and use "were" because each variable has several items. | | In line with this comment, the number of items used to measure each variable has been determined. [Location of change: Section 3.2, page 7]. |
| - 4.1 Measurement model (start with the capital letter). | | According to your suggestion, the title has been corrected, with a capital letter at the beginning of the word “Measurement”. [Location of change: Section 4.1, page 8]. |
| - The name of Table should be changed to "Direct and Moderation Effect" to suit with the info. in the table. | | Based on your suggestion, the name of Table 3 has been changed to become “Direct and Moderation Effect”. [Location of change: Section 4.2, page 10]. |
| - Under Table 5, correct how to write Q-Square in Line 3 (Q2 is incorrect since it can lead to misunderstand as Second Quartile) | | Based on your feedback, the abbreviation (Q-Square) has been corrected to avoid misunderstandings. [Location of change: Section 4.2, page 11]. |
| - The name of Figure 4 "Interactive effect of BCT and ED on SCI" is incorrect because the figure explains the moderation effect of ED on the path linking BCT and SCI. (Suggested Figure Name: "Moderation Effect of ED on BCT and SCI") | | Based on your suggestion, the name of Figure 4 has been changed to become “Moderation Effect of ED on BCT and SCI”. [Location of change: Section 4.2, page 11]. |

| **Responses to Reviewer 2** | |
| --- | --- |
| **Comment** | **Response** |
| Dear Reviewer,  Thank you very much for your encouraging words which greatly motivate us to further improve the quality of our research paper. Moreover, thank you very much for your review of our work and the provision of your constructive feedback. Following your suggestions, we have substantially revised our manuscript to incorporate your valuable and detailed feedback.  Best regards | |
| The writing style is very good and the topic is very interested and will attract the reader who interest in the block chain technology and the supply chain resilient. The reference list is quite update but if you can update some of the reference list to the newer one, your paper will be more interesting. | Many thanks for your suggestions. Accordingly, some of the references have been updated. |
